# Dr. O. W. Wight

**Published:** 1881-12

**Authors:** 


Dr. O. W. Wight, of Milwaukee, one of the most thoroughly competent, practical sanitarians in the country, has been elected health officer of Detroit, Mich. This is one of the few instances in which competence has triumphed over local political influence. The city of Detroit is to be congratulated on this practical example of reform in the civil service.

The Graduating Exercises of the medical department of Dartmouth College were held on Nov. 14th, 1881. The graduating class numbered thirty-six.

